# Rapid MRI of the lungs in children with pulmonary infections

**DOI:** 10.1590/0100-3984.2016.0025

**Published:** 2016

**Authors:** Kushaljit Singh Sodhi, Anmol Bhatia, Niranjan Khandelwal

**Affiliations:** 1 Department of Radiodiagnosis and Imaging, Post Graduate Institute of Medical Education and Research, Sector-12, Chandigarh 160012, India.; 2 GE Radiology Section, Department of Gastroenterology, Post Graduate Institute of Medical Education and Research, Sector-12, Chandigarh 160012, India.

*Dear Editor*,

We read with interest the review article entitled "Chest magnetic resonance imaging: a protocol suggestion" by Hochhegger et al.^(1)^. The authors have reviewed the technical aspects and suggested a protocol for performing chest MRI. The authors have also described three major clinical indications for MRI of the lungs: staging of lung tumors; evaluation of pulmonary vascular diseases; and investigation of pulmonary abnormalities in patients who should not be exposed to radiation. Radiation exposure is particularly more serious in children, as they are at a greater risk of experiencing harmful effects from radiation compared to adults^(2)^.

In our recent prospective study in 26 children with leukemia presenting with febrile neutropenia^(3)^, we evaluated role of rapid lung MRI in the detection of nodules, consolidation and ground glass opacity (GGO) in this population. The duration of all the four sequences combined in our study was less than 2 minutes. The findings of HRCT and MRI were compared, with HRCT as the standard of reference. No significant difference was observed between the two modalities by the McNemar test (p > 0.05). For the detection of nodules and consolidation by MRI, per-patient sensitivity, specificity, positive predictive value (PPV) and negative predictive value (NPV) were all 100%. For the detection of GGO by MRI, per-patient sensitivity, specificity, PPV and NPV were 66.67%, 100%, 100% and 90.91%, respectively. The kappa test showed perfect agreement between MRI and CT scan for the detection of nodules and consolidation (κ = 1), and a substantial agreement in the detection of GGO by MRI and CT scan (κ = 0.755). The results of our study indicated that pulmonary MRI has great potential as a diagnostic modality for the detection of lung parenchymal findings in patients with febrile neutropenia.

Similarly, we determined the diagnostic utility of rapid lung MRI for the detection of various pulmonary and mediastinal abnormalities in 75 children with suspected pulmonary infections^(4)^. MRI demonstrated sensitivity, specificity, PPV, and NPV of 100% for detecting pulmonary consolidation, nodules (> 3 mm), cyst/ cavity, hyperinflation, pleural effusion, and lymph nodes. The kappa test showed almost perfect agreement between MRI and MDCT in detecting thoracic abnormalities (κ = 0.965). No statistically significant difference was observed between MRI and MDCT for detecting thoracic abnormalities by the McNemar test (p = 0.125).

As MRI does not have any radiation risks, it can be repeated to assess disease progression or regression without exposing the patients to radiation (as against performing the CT scan). We propose rapid lung MRI may also be used as an initial radiological investigation in patients with suspected pulmonary infections especially where repeated follow up imaging is required.
